# COVID-19 and stem cell transplantation; results from an EBMT and GETH multicenter prospective survey

**DOI:** 10.1038/s41375-021-01302-5

**Published:** 2021-06-02

**Authors:** Per Ljungman, Rafael de la Camara, Malgorzata Mikulska, Gloria Tridello, Beatriz Aguado, Mohsen Al Zahrani, Jane Apperley, Ana Berceanu, Rodrigo Martino Bofarull, Maria Calbacho, Fabio Ciceri, Lucia Lopez-Corral, Claudia Crippa, Maria Laura Fox, Anna Grassi, Maria-Jose Jimenez, Safiye Koçulu Demir, Mi Kwon, Carlos Vallejo Llamas, José Luis López Lorenzo, Stephan Mielke, Kim Orchard, Rocio Parody Porras, Daniele Vallisa, Alienor Xhaard, Nina Simone Knelange, Angel Cedillo, Nicolaus Kröger, José Luis Piñana, Jan Styczynski

**Affiliations:** 1 Department of Cellular Therapy and Allogeneic Stem Cell Transplantation, Karolinska Comprehensive Cancer Center, Karolinska University Hospital Huddinge, Stockholm, Sweden; 2 Division of Hematology, Department of Medicine Huddinge, Karolinska Institutet, Stockholm, Sweden; 3 Department of Hematology, Hospital de la Princesa, Madrid, Spain; 4 Division of Infectious Diseases, University of Genoa and Ospedale Policlinico San Martino, Genova, Italy; 5 Pediatric Hematology Oncology, Verona, Italy; 6 King Abdul – Aziz Medical City, Riyadh, Saudi Arabia; 7 Centre for Haematology, Imperial College, London, UK; 8 Hopital Jean Minjoz, Besancon, France; 9 Hospital Santa Creu i Sant Pau, Barcelona, Spain; 10 Hospital Universitario 12 de Octubre, Madrid, Spain; 11 Ospedale San Raffaele s.r.l., Milan, Italy; 12 Hematology Department, Complejo Asistencial Universitario de Salamanca-IBSAL; Centro de Investigación del Cáncer-IBMCC, Salamanca, Spain; 13 Spedali Civili, Brescia, Italy; 14 Servei d’Hematologia, Vall d’Hebron Hospital Universitari, Experimental Hematology, Vall d’Hebron Institute of Oncology (VHIO), Vall d’Hebron Barcelona Hospital Campus, Barcelona, Spain; 15 ASST Papa Giovanni XXIII, Bergamo, Italy; 16 ICO-Hospital Germans Trias i Pujol. Josep Carreras Research Institute, Badalona, Spain; 17 HSCT unit, Demiroglu Bilim University, Istanbul, Turkey; 18 Hospital General Universitario Gregorio Marañon, Instituto de Investigación sanitaria Gregorio Marañon, Madrid, Spain; 19 Hospital Universitario Donostia, San Sebastian, Spain; 20 Fundación Jiménez Díaz, Madrid, Spain; 21 Department of Laboratory Medicine, Karolinska Institutet, Stockholm, Sweden; 22 Southampton General Hospital, Southampton, UK; 23 ICO – Hospital Duran i Reynals, Barcelona, Spain; 24 Hospital Guglielmo da Saliceto, Piacenza, Italy; 25 Hôpital St. Louis, Paris, France; 26 EBMT Data Office, Department of Medical Statistics & Bioinformatics, Leiden, Netherlands; 27 GETH Spanish Registry, Madrid, Spain; 28 Department of Stem cell Transplantation, University Hospital Eppendorf, Hamburg, Germany; 29 Department of Hematology, Hospital Clinico Universitario de Valencia. Fundación de investigación INCLIVA, Hospital Clinico Universitario de Valencia, Valencia, Spain; 30 Pediatric Hematology and Oncology, University Hospital, Collegium Medicum, Nicolaus Copernicus University Torun, Bydgoszcz, Poland

**Keywords:** Infectious diseases, Haematological diseases

## Abstract

This study reports on 382 COVID-19 patients having undergone allogeneic (*n* = 236) or autologous (*n* = 146) hematopoietic cell transplantation (HCT) reported to the European Society for Blood and Marrow Transplantation (EBMT) or to the Spanish Group of Hematopoietic Stem Cell Transplantation (GETH). The median age was 54.1 years (1.0–80.3) for allogeneic, and 60.6 years (7.7–81.6) for autologous HCT patients. The median time from HCT to COVID-19 was 15.8 months (0.2–292.7) in allogeneic and 24.6 months (−0.9 to 350.3) in autologous recipients. 83.5% developed lower respiratory tract disease and 22.5% were admitted to an ICU. Overall survival at 6 weeks from diagnosis was 77.9% and 72.1% in allogeneic and autologous recipients, respectively. Children had a survival of 93.4%. In multivariate analysis, older age (*p* = 0.02), need for ICU (*p* < 0.0001) and moderate/high immunodeficiency index (*p* = 0.04) increased the risk while better performance status (*p* = 0.001) decreased the risk for mortality. Other factors such as underlying diagnosis, time from HCT, GVHD, or ongoing immunosuppression did not significantly impact overall survival. We conclude that HCT patients are at high risk of developing LRTD, require admission to ICU, and have increased mortality in COVID-19.

## Introduction

A novel coronavirus named SARS-CoV-2 emerged at the end of 2019, and Coronavirus Disease 2019 (COVID-19) started spreading worldwide [1–3]. The WHO classified COVID-19 a pandemic on March 11, 2020, and as of February 8, 2021, more than 106 million cases have been verified worldwide, and more than 2,300,000 have died. The EBMT has published recommendations regarding polices and patient management [4]. There have been major achievements in the management of COVID-19, but the mortality remains high especially in patients with risk factors. Recently, extensive vaccination programs were initiated to control transmission and to reduce morbidity and mortality.

Hematopoietic cell transplant recipients (HCT) are prone to develop severe infections with many viruses including community-acquired respiratory viruses. To better understand the impact of COVID-19, data collection began via the Infectious Diseases Working Party of the EBMT and the Spanish Hematopoietic Stem Cell Transplantation and Cell Therapy Group (GETH). This manuscript presents the first results of this collaborative effort.

This paper aimed to analyze the outcome of the first wave of COVID-19 for patients registered in the study and identified risk factors for lower respiratory tract disease (LRTD), the requirement for intensive care, and mortality.

## Methods

This is an EBMT and GETH prospective survey merging newly collected data with previous data existing in the EBMT registry. All patients give informed consent for their data to be included in the registry. Questions included the symptoms at the time of diagnosis, potential risk factors for development of pneumonia, the need for hospitalization, intensive care, and outcome. Criteria for inclusion in the study were that the patient should be PCR positive for SARS-CoV-2 regardless of symptoms and have undergone an allogeneic or autologous HCT at any time before the diagnosis of COVID-19. A parallel data collection with forms written in Spanish was used to collect data from Spain by GETH. The two sets of case record forms (CRFs) were almost identical with a few additional data fields used in the Spanish forms. The Swedish central Ethical Board (EPM 2020-01731) approved the study and other approvals if required, were obtained according to national regulations.

For this analysis, patients diagnosed with SARS-CoV-2 infection at or before July 31, 2020, were included and patients needed to have at least 6 weeks of follow-up. In addition to the COVID-19 specific forms, the EBMT registry’s so-called Minimal Essential Data A (MED-A) was used to extract previously submitted data regarding baseline patient information, data regarding the underlying diagnosis, and the transplant procedure, which were used in the analysis.

### Definitions

#### Resolution

The resolution was defined as being alive with either clinical or virologic resolution of COVID-19.

#### Lower respiratory tract disease

Since signs of LRTD are diverse and may develop over time, several questions in the CRFs (i.e., oxygen support requirements, pulmonary radiology findings, the results of bronchoalveolar lavage, and the presence of clinical signs of LRT such as shortness of breath, sibilants, rales, and cough), were used to define LRTD. This assessment was performed by two of the investigators (JLP and PL) and used for the analysis.

#### Immunodeficiency scoring index (ISI)

This was calculated as previously described [5]. This score was prepared for allogeneic HCT patients. Still for the purpose of this study we used it also to analyze the complete population as well as allogeneic and autologous separately.

Absolute lymphocyte count/C-reactive protein (ALC/CRP) ratio was defined according to Lagunas-Rangel, 2020 [6].

### Statistics

The main characteristics of patients were reported by descriptive statistics. Median, minimum and maximum values were used for continuous variables, while absolute and percentage frequencies were used for categorical variables. The overall survival was estimated by using the Kaplan–Meier methods, considering the death due to any cause as an event and the time from COVID-19 infection to the latest follow-up as survival time; the difference between groups was tested by the log-rank test. Univariate and multivariate risk factor analysis for overall survival were performed with the Cox regression model. Univariate and multivariate risk factor analyses for COVID-19 resolution were performed with the cause-specific Cox regression model, considering the resolution as the event of interest and death due to any cause as a competing event. Univariate and multivariate risk factor analyses for the requirement of admission to an intensive care unit (ICU) and LRTD development were performed with the logistic regression model. Variables with a *p* value <0.2 at univariate analysis were entered into the multivariate models and selected according to a stepwise selection. A *p* value <0.05 was considered statistically significant. All *p* values are two-sided. All the analyses were performed using the statistical software SAS v. 9.4 (SAS Institute Inc., Cary, NC, USA).

## Results

### Number of included patients

Three hundred eighty-two patients from 22 countries fulfilled the criteria for inclusion. Two hundred thirty-six patients had undergone allogeneic HCT and 146 autologous. The median age in the entire cohort was 56.8 years (min–max; 1.0–81.6). The corresponding median ages were 54.1 years (min–max; 1.0–80.3) for allogeneic and 60.6 years (7.7–81.6) for autologous HCT patients, respectively. Thirty-two patients were children (<18 years of age; median age 9.5 (1.0–16.9)). The median time from the most recent HCT to diagnosis of COVID-19 was 17.9 months (min–max; −0.9 to 350.3). The corresponding median times were 15.8 months (min–max 0.2–292.7) in allogeneic and 24.6 months (min–max; −0.9 to 350.3) in autologous HCT recipients, respectively.

Additional patient characteristics are shown in supplementary Table 1. Details about donor type, conditioning, stem cell source, and GVHD for the patients having undergone allogeneic HSCT are shown in Table 1. 74.4% of the patients were hospitalized during the COVID-19 episode.

**Table 1:** Characteristics of patients having undergone allogeneic HCT.

	*N* = 236
Stem cell source	
BM (bone marrow)	37 (15.7)
PB (peripheral blood)	186 (78.8)
CB (cord blood)	4 (1.7)
Missing	9 (3.8)
HLA match	
Matched family	78 (33.0)
Unrelated	111 (47.0)
Mismatched family	35 (14.8)
Missing	12 (5.1)
Conditioning	
Myeloablative	108 (45.8)
Reduced	111 (47.0)
Missing	17 (7.2)
In-vivo T-cell depletion	
No	109 (46.2)
Yes	121 (51.3)
Missing	6 (2.5)
aGvHD at time of COVID-19	
no aGvHD/grade 1	132 (55.9)
grade 2–4	12 (5.1)
Chronic GvHD at time of COVID-19	
No (never)	137 (58.0)
Yes, ongoing	77 (32.6)
Resolved	12 (5.1)
Missing	10 (2.8)
Corticosteroids	
No	133 (56.3)
Yes	88 (37.3)
Missing	15 (6.3)

### Symptoms at diagnosis

Symptoms at diagnosis are summarized in Supplementary Table 2. Thirty-four patients (8.9%) were reported to be asymptomatic during SARS-CoV-2 infection. On the other hand, 132/376 patients (35.1%) required supplemental oxygen to keep saturation above 92% (data missing for six patients). The median time to diagnosis from the start of symptoms was 3 days (from thirty days before to 40 days after the onset of symptoms).

### Interventions

Different interventions were attempted in this patient cohort reflecting the knowledge at the time of COVID-19. These are summarized in Supplementary Table 3. Data is not available regarding the use of anticoagulation as prophylaxis against or treatment of thromboembolic events.

### Outcome of COVID-19

At the time of analysis, 107/377 (28.4%) patients had died (66/231 allogeneic; 41/146 autologous). 69/236 (29.2%) of males and 38/146 (26.0%) of females had died at time of analysis (*p* = 0.48). The corresponding percentages in allogeneic HCT recipients were 28.5% vs. 27.2% in males and females, respectively. The median age of patients, who died, was 62.2 years (4.5–80.3). Twelve patients died from other causes than COVID-19 giving an attributable mortality from COVID-19 of 25.2%. No follow-up had been received for five patients. The median time from confirmed infection to death was 18 days.

Thirty-two children were included in the study: 29 after allogeneic and three after autologous HCT. Three of 32 children died: all after allogeneic HCT. The median time from HCT to COVID-19 diagnosis in the children, who died, was 2 months (2–15) while it was 4 months (−1 to 56) in the children surviving COVID-19. Furthermore, there was no difference in age between the children, who died (8 years; 4–12) or survived (10 years; 1–17).

### Overall survival

**Fig. 1:**
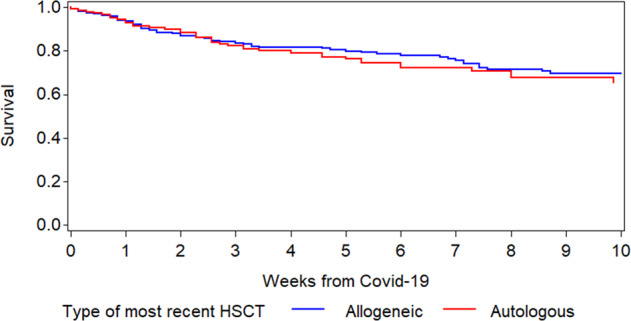
Overall survival after diagnosis of COVID-19 infection in allogeneic and autologous HCT recipients.

**Fig. 2:**
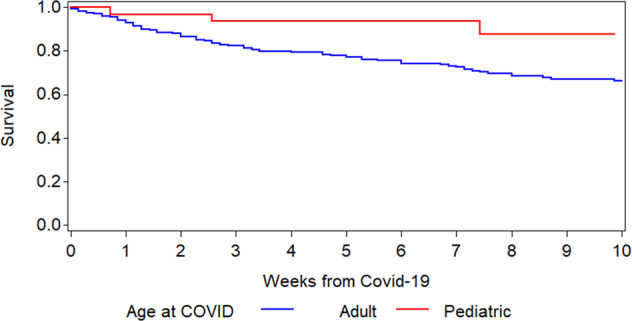
Survival after diagnosis of COVID-19 in adults and children.

The Kaplan–Meier overall survival curves for allogeneic and autologous HCT patients are shown in Fig. 1. The probabilities for survival at 6 weeks from diagnosis of COVID-19 were 77.9% and 72.1% in allogeneic and autologous HCT recipients, respectively. There was no significant difference in survival between autologous and allogeneic HCT patients (*p* = 0.8). Children had a higher survival rate (*p* = 0.03; Fig. 2).

In univariate analysis (Supplementary Table 4), risk factors influencing overall survival in the total population were age (*p* < 0.0001), performance status (*p* < 0.0001), time from HCT to COVID-19 (*p* = 0.04), ISI intermediate + high vs. low (*p* = 0.0008), ANC (*p* = 0.018), and the ALC/CRP ratio (*p* = 0.009). The age effect also remained when only adults were included in the analysis. In addition, the need for admission to ICU had a very strong impact on overall survival (*p* < 0.0001). Looking more in detail on time from HCT to COVID-19, the mortality in patients developing COVID-19 the first 100 days after HCT was 35.8% vs. 26.8% (*p* = NS) in those developing COVID-19 beyond 100 days from HCT. The distribution of times from HCT to diagnosis of COVID-19 and mortality is shown in Table 2. The outcome in patients on or off immunosuppression split by time from HCT to COVID-19 is shown in Supplemenary Fig. 1.

**Table 2:** Impact of time between HCT and diagnosis of COVID-19 on mortality.

	Total		Allo		Auto	
Time after HCT	Patients	Deaths	Patients	Deaths	Patients	Deaths
0–100	67	24	44	17	23	7
100-6 mm	31	9	22	7	9	2
6mm-1y	56	19	37	12	19	7
12–18 mm	36	12	24	10	12	2
18mm-2y	26	6	18	2	8	4
≥2yy	161	37	89	18	72	19

In multivariate analysis (Table 3), age (HR 1.21; 95% CI 1.03–1.43; *p* = 0.02) and higher ISI group (HR 1.84; 95% CI 1.02–3.33; *p* = 0.04) increased the risk and better performance status decreased the risk for fatal outcome (HR 0.83; 95% CI 0.74–0.93; *p* = 0.0001). These factors remained significant also when adjusting for type of transplantation and if admission to ICU was not included as a variable in the analysis.

**Table 3:** Multivariate analysis for risk factors for different analyzed outcomes.

Variable	Characteristics	Overall survival		COVID-19 resolution		LRTD		ICU admission	
		HR (95% CI)	*p*	HR (95% CI)	*p*	OR (95% CI)	*p*	OR (95% CI)	*p*
Age at COVID-19	Continuous (10-yr effect)	1.21 (1.03–1.43)	0.02		ns	1.41 (1.17–1.69)	0.0002	–	ns
Time from most recent transplant to COVID-19	≥1 year vs <1 year	–	ns		ns	–	ns		ns
Performance status	Continuous (10-point effect)	0.83 (0.74–0.93)	0.001	1.16 (1.02–1.32)	0.02	–	ns	–	ns
ICU admission	Yes	3.17 (2.00–5.01)	<0.0001	0.36 (0.22–0.60)	<0.0001		ns		ns
ISI group	Moderate /high	1.84 (1.02–3.33)	0.04					2.59 (1.33–5.05)	0.005
ALC/CRP^a^	<median vs > median		ns		hs	0.32 (0.13–0.80)	0.015		hs
Underlying disease	AML/ALL								
	CML/MDS/MPN								
	NHL/Hodgkin/ CLL		ns		ns		ns		ns
	Other								
Country	Spain							1.00	
	Italy–UK		ns		ns		ns	0.94 (0.42–2.12)	0.007
	Other							2.51 (1.31–4.80)	
Allo only									
Age at COVID-19	Continuous (10-yr effect)	1.29 (1.05–1.58)	0.01	–	ns	1.39 (1.14–1.71)	0.002	–	ns
Time from most recent transplant to COVID-19	≥1 year vs <1 year	–	ns	–	ns	–	ns	–	ns
Performance status	Continuous (10 point effect)		ns	1.20 (1.07–1.36)	0.003	–	ns		ns
ICU admission	Yes	4.42 (2.25–8.65)	<0.0001	0.37 (0.24–0.59)	<0.0001		ns		
Current IS therapy	Yes		ns		ns	2.78 (1.02–7.60)	0.046	3.69 (1.06–12.87)	0.04
Country	Spain			1.00					
	Italy-UK		ns	1.20 (0.81–1.80)	0.009		ns		ns
	Other			1.70 (1.20–2.40)					
Auto only									
Age at COVID-19	Continuous (10-yr effect)	2.26 (1.22–4.20)	0.01	–	ns	1.72 (1.07–2.74)	0.02	–	ns
ICU admission	Yes			0.48 (0.32–0.71)	0.0002				
Time from most recent transplant to COVID-19			ns		ns	3.93 (1.38–11.22)	0.01		ns

*ISI* immunodeficiency scoring index, *ICU* intensive care unit.

^a^*ALC/CRP* absolute lymphocyte count/CRP ratio.

Factors significant in univariate analysis for overall survival in allogeneic HCT recipients were age (*p* = 0.0007), performance status (*p* < 0.0001), need for admission to ICU (*p* < 0.0001), and ISI group intermediate or high (*p* = .0.0005). The age effect also remained when only adults were included in the analysis. In multivariate analysis, excluding ICU admission age (HR 1.28; 95% CI 1.05–1.55; *p* = 0.01) and performance status (HR 0.79; 95% CI 0.68–0.92; *p* = 0.002) had a significant impact on overall survival. Only age (HR 1.29; 95% CI 1.05–1.58; *p* = 0.01) remained significant when ICU (HR 4.42; 95% CI 2.25–8.65; *p* < 0.0001) was included in the model.

The factors significant in univariate analysis for overall survival in autologous HCT patients were age (*p* = 0.01), performance status (*p* = 0.02), and admission to ICU (*p* = 0.002). The age effect also remained when only adults were included in the analysis. The only factor impacting overall survival in multivariate analysis (Table 3) was higher age (HR 2.26; 95% CI 1.22–4.20; *p* = 0.01).

### Resolution

Of the patients reported to be alive, the median follow-up was 47 days (5–250 days). One hundred eighty-six (64.3%) patients (117 allogeneic; 69 autologous), excluding patients who died from COVID-19, had virologic resolution of the COVID-19 infection. The median time to virologic resolution was 24 days with the longest being 210 days. In addition, 58 patients (37 allogeneic and 21 autologous) had clinical resolution but had not been tested again with PCR. 25 patients were alive but known to be still COVID-19 positive (13 allogeneic; 12 autologous). We had no information on five patients.

Factors impacting on resolution were in univariate analysis high-performance status (1.14 (1.02–1.27), *p* = 0.02), moderate/high ISI group (0.73 (0.54–0.99), *p* = 0.04), lung pathology (0.70 (0.48–1.01), *p* = 0.05), the underlying diagnosis (NHL/Hodgkin/CLL vs. AML/ALL 0.65 (0.45–0.94), *p* = 0.02), and ALC ≥ 200 (1.64 (1.02–2.66), *p* = 0.04; supplementary table 4). Besides, ICU admission impacted on resolution (0.47 (0.32–0.70), *p* = 0.0002). In multivariate analysis (Table 3), only the underlying disease had a significant impact if ICU was not in the model, while ICU admission and performance status were significant in a model including ICU care.

In allogeneic HCT patients the factors influencing resolution in univariate analysis were high-performance status (1.24 (1.08–1.43), *p* = 0.003), ISI group moderate/high (0.58 (0.39–0.84), *p* = 0.004), presence of GVHD (0.70 (0.50–0.99), *p* = 0.04), ALC ≥ 200 (2.03 (1.08–3.83), *p* = 0.03), need for ICU care (0.42 (0.25–0.70), *p* = 0.001), and ongoing immunosuppressive treatment (0.57 (0.39–0.83), *p* = 0.003; Supplementary Table 4). In multivariate analysis the underlying diagnosis excluding the ICU variable, time from HCT to COVID-19, the diagnosis, and ISI group influenced the time to resolution while with ICU variable included, ICU admission, performance status and country-influenced resolution (Table 3).

### Lower respiratory tract disease (LRTD)

308/369 (83.5%) patients developed LRTD (data missing for 13 patients). Results of the univariate analyses are shown in Supplementary Table 1. In multivariate analysis (Table 3), increasing age (HR 1.41; 95% CI 1.17–1.69; *p* = 0.0002) and lymphocyte/CRP ratio (HR 0.32; 95% CI 0.13–0.80; *p* = 0.015) were significant in the total population. In allogeneic HCT patients, age (HR 1.39; 95% CI 1.14–1.71; *p* = 0.002) and ongoing immunosuppression (HR 2.78; 95% CI 1.02–7.60; *p* = 0.046) increased the risk for LRTD while in autologous patients age (HR 1.72; 95% 1.07–2.74; *p* = 0.02) and longer time after HCT (HR 3.93; 95% 1.38–11.22; *p* = 0.01) had a significant impact on the risk for LRTD.

### Requirement of admission to an intensive care unit

80/356 (22.5%) patients received ICU care (data missing for 26 patients). The median time from diagnosis of COVID-19 to admission to ICU among 69 patients for whom we have information (data missing on 11 patients) was five days (−5 to 35). The median time from diagnosis to ICU care was seven days in allogeneic and four days in autologous HCT patients. 38/69 patients died within six weeks from the day of ICU admission.

Patients diagnosed with COVID-19 later than one year after HCT had in univariate analysis (Supplementary table 4) a lower risk of admission to an ICU (*p* = 0.02). Other factors are shown in Supplementary Table 1. In multivariate analysis, moderate/high ISI group (OR 2.59; 95% CI 1.33–5.05; *p* = 0.005) and country (*p* = 0.007) were significant factors for ICU admission. If ISI was not included in the model, the ALC/CRP ratio was significant together with country (Table 3).

In multivariate analysis including only allogeneic HCT recipients, only ongoing immunosuppression increased the risk for ICU admission (HR 3.69; 95% CI 1.06–12.87; *p* = 0.04). No factor had significant impact on the risk for admission to ICU among autologous patients in multivariate analysis.

## Discussion

The emergence of SARS-CoV-2 and COVID-19 has had a very strong impact on transplant centers fearing for the outcome of this disease in a population known to be vulnerable to viral infections in general and to community-acquired respiratory viruses [7–12]. Here, we present a series of 382 patients from 22 countries collected prospectively through the EBMT registry and in close collaboration with the Spanish GETH, which is the largest patient cohort with COVID-19 after HCT presented to date.

Overall, COVID-19 was a severe complication in HCT recipients with an attributable mortality of 25%. This can be compared to the case fatality rates during the 1st wave in some European countries and the USA, 4–10% (https://www.worldometers.info/coronavirus/). The median age in our population of patients having undergone allogeneic HCT was 54.1 years and 60.6 years in patients having undergone autologous HCT, with the majority being below 70 years of age. We found a 6-week probability of survival of 77.9% and 72.1% in allogeneic and autologous HCT recipients, respectively. The mortality rates in Sweden were 4.6%, 1.1%, and 0.1% in patients 60–69, 50–59, and <50 years of age, respectively (www.folkhalsomyndigheten.se/ August 13, 2020). This date corresponds approximately with the cut-off date for inclusion in this patient series. Thus, the mortality in our cohort is much higher than in the general population. In the general population, mortality seems to be lower in the second wave and it remains to be seen if that is the case in HCT recipients as well.

The CIBMTR published results on 314 patients with COVID-19 [13]. Their 30-day survival after diagnosis of COVID-19 was 68% among allogeneic HCT recipients and 67% among autologous HCT recipients. They found age over 50, male sex, and time from HCT to COVID-19 diagnosis of less than a year to be significant risk factors for mortality among allogeneic recipients. In contrast, only diagnosis of lymphoma had an impact on survival among autologous recipients. They did not report the impact performance status at COVID-19 as a potential risk factor, which was highly significant in our series. On the other hand, we could not find any impact of sex on mortality. They reported only 10.9% mortality among female allogeneic HCT recipients compared to 27.2% in our series. The corresponding percentages in males were 33.7% vs. 28.5% in the CIBMTR and our series, respectively. These differences are unexplained.

In another smaller series, Shah et al. reported a 30-day survival of 78% in 72 HCT patients and 5 CAR T-cell-treated patients [14]. Their median time from HCT to COVID-19 diagnosis was slightly more than 2 years compared to 18 months in our study. Although in our cohort, in multivariate analysis, time from HCT to COVID-19 was not an independent risk factor for poor outcome, the mortality was 36% in patients diagnosed the first 100 days after HCT. Coll et al. reported in a series, which included solid organ transplant patients [15], a mortality of 20% and 24% among 56 allogeneic HCT and 29 autologous HCT patients, respectively. In this study, the time median from HCT to COVID-19 was similar as in our study (15 months in allogeneic and 18 months in autologous HCT recipients). Other studies of HCT patients have reported similar outcomes [14–22].

Cancer patients are more vulnerable to develop severe COVID-19. In a study of 107 cancer patients in China with a median age of 66 years, the mortality was 21.5% [3], which is comparable to our patient cohort. However, our cohort’s median age was a decade younger. Scarfò et al reported high mortality in patients with CLL with a median age of 72 years and found that the risk for severe COVID-19 disease was associated with higher age (>65 years). Still, neither older age nor comorbidities influenced mortality [23]. In a series of solid organ transplanted patients, Kates et al. reported a similar mortality of 20.5% at 28 days after diagnosis of infection [24].

Other indicators of the severity of COVID-19 in our patients were a rate of LRTD of 83.5% and a rate of admission to ICU of 22.5%. These figures can be compared to the experience with the H1N1 “Swine flu” pandemic in which the corresponding numbers were 32.5% and 11.5% for LRTD development and need of intensive care, respectively [25]. The figures can also be compared to the rates of ICU admission in Sweden during the 1st wave, which is a country where there was no lack of ICU beds during the pandemic. Given that the median age of the HCT cohort was 56 years the best corresponding rates of ICU admission in Sweden were 8.9% in patients 60–69 years, 4.3% in those 50–59 years, and 1.1% in patients below 50 years of age (www.folkhalsomyndigheten.se/ August 13, 2020). Nine percent of our cases were asymptomatic, but it is likely that our numbers reflect a selection bias since not all patients having undergone HCT and infected with SARS-CoV-2 have sought medical care, especially those with no or mild symptoms or those being a long time after HCT and without complications from the transplantation. Additional studies of serological evidence of COVID-19 might elucidate this question.

We calculated indices to see if any of those helped predict outcome. ISI impacted on survival and ICU admission in the total population. It also impacted on resolution and ICU admission in patients having undergone allogeneic HCT but had no added value in autologous patients. This index was developed for allogeneic HCT with RSV infections. This report supports that it is also useful for COVID-19. The ALC/CRP ratio also had a significant impact on the risk for LRTD and need for ICU admission but not for survival. Both these are easy to calculate and might be helpful in the clinical assessment of SARS-CoV-2 patients.

Some of our findings were expected such as that overall survival was influenced by patient age and performance status. Age has been an important factor in most reports on COVID-19 in the general population [26–28]. Poor performance status is usually due to comorbidities such as extensive GVHD, but in this cohort, we were unable to find a correlation of specific comorbidities with the HCT outcomes. Kates et al. also found age and comorbid conditions as drivers of mortality in solid organ transplant recipients [24]. Children seemed to do better. We present results of COVID-19 in 32 children after HCT of whom 29 survived. This also fits with what has been reported in the general population [29], and also with a report of eight HCT children with COVID-19 of whom seven survived [17].

More surprising was that the type of transplantation did not impact on overall survival. Allogeneic HCT patients are much more likely to develop severe complications from other respiratory viruses such as RSV and parainfluenza viruses [30–32]. However, studies reported no difference between risk for mortality in autologous and allogeneic HCT patients with influenza such as during the H1N1 “Swine flu” pandemic [25, 33].

It was also surprising that the time from HCT did not impact overall survival and neither did ongoing immunosuppression, presence of GVHD, preexisting pulmonary morbidity, or the granulocyte or lymphocyte count. Several of these factors have been shown to be of importance for outcome in other respiratory virus infections [5, 7, 12, 34, 35]. Contracting SARS-CoV-2 infection later than a year after allogeneic HCT was associated with a higher rate of resolution. Furthermore, when using the ISI, which includes several of these factors to create an assessment of the total immunodeficient status [5], patients with a moderate or high index had increased mortality if ICU admission was not included as a variable. Furthermore, high ISI increased the patient’s risk to require ICU care and negatively impacted on time to resolution among allogeneic HCT recipients. In addition, ongoing immunosuppressive therapy impacted the risk for LRTD and the need for ICU admission in allogeneic HCT recipients. An essential factor for the morbidity in COVID-19 is immune system activation including effects on the T-cell responses [36–38]. It could be that patients on immunosuppression might be partly protected against this aspect of COVID-19 pathogenesis. In contrast, immunosuppression could prolong viral excretion by suppressing viral clearance either by the production of specific antibodies or by suppressing the T cell or NK cell functions. These possibilities will have to be investigated in a larger cohort.

It should be recognized that this study included patients developing COVID-19 during the 1st wave. Although we have information on used therapeutic interventions, the data do not allow detailed analysis of the potential effects of antiviral drugs or anti-inflammatory agents due to the heterogeneity of the population, the non-controlled nature of the study, and the variation in different interventions making it too difficult, even with the size of this study, to get reliable estimates of potential therapeutic effects.

We can conclude from this study that COVID-19 resulted in high rates of morbidity and mortality supporting that preventing infections in these patients is of uttermost importance. Furthermore, even in a high-risk cohort such as HCT recipients, there are groups at higher and lower risk for poor outcome. The most critical factors are older age and poor performance status presumably due to comorbidities although we could not pinpoint important individual comorbidities. Hopefully, this knowledge can help patients and transplant centers to design interventions and protective measures including vaccines to decrease this risk in this highly vulnerable patient group.

## Supplementary information

Supplementary tables and figure

Contributors
