# Supplementary material for: COVID-19 and stem cell transplantation; results from an EBMT and GETH multicenter prospective survey
Source: Leukemia. 2021 Jun 2;35(10):2885–94. doi: 10.1038/s41375-021-01302-5 (PMC8171362; doi:10.1038/s41375-021-01302-5)

Supplementary tables and figure.

Supplementary table 1: Patient characteristics.

		Type of most recent HCT		Total (N=382)
		Allogeneic	Autologous	
		N (%)	N (%)	N (%)
Country	Spain	72 (30.5)	67 (45.9)	139 (36.4)
	United Kingdom	32 (13.6)	25 (17.1)	57 (14.9)
	Italy	30 (12.7)	16 (11.0)	46 (12.0)
	France	19 (8.1)	9 (6.2)	28 (7.3)
	Sweden	20 (8.5)	1 (0.7)	21 (5.5)
	Belgium	10 (4.2)	5 (3.4)	15 (3.9)
	Netherlands	7 (3.0)	5 (3.4)	12 (3.1)
	Turkey	9 (3.8)	3 (2.1)	12 (3.1)
	Saudi Arabia	6 (2.5)	4 (2.7)	10 (2.6)
	Germany	9 (3.8)	0 (0.0)	9 (2.4)
	Portugal	3 (1.3)	3 (2.1)	6 (1.6)
	Switzerland	3 (1.3)	1 (0.7)	4 (1.0)

		Type of most recent HCT		Total
		Allogeneic	Autologous	(N=382)
		N (%)	N (%)	N (%)
	Israel	2 (0.8)	2 (1.4)	4 (1.0)
	Iran	4 (1.7)	0 (0.0)	4 (1.0)
	Denmark	3 (1.3)	0 (0.0)	3 (0.8)
	Czech Republic	3 (1.3)	0 (0.0)	3 (0.8)
	Algeria	0 (0.0)	3 (2.1)	3 (0.8)
	Greece	0 (0.0)	1 (0.7)	1 (0.3)
	Norway	1 (0.4)	0 (0.0)	1 (0.3)
	Poland	1 (0.4)	0 (0.0)	1 (0.3)
	Ireland	1 (0.4)	1 (0.7)	2 (0.5)
	Russia	1 (0.4)	0 (0.0)	1 (0.3)
Gender	Male	144 (61.0)	92 (63.0)	236 (61.8)
	Female	92 (39.0)	54 (37.0)	146 (38.2)

		Type of most recent HCT		Total
		Allogeneic	Autologous	(N=382)
		N (%)	N (%)	N (%)
Diagnosis	Acute myeloid leukemia	75 (31.8)	5 (3.4)	80 (20.9)
	Plasma cell disorders	10 (4.7)	82 (56.2)	92 (24.1)
	Non-Hodgkin lymhoma	21 (8.9)	43 (29.4)	64 (16.8)
	Myelodysplastic syndromes / myeloproliferative disorders	53 (22.4)	0 (0.0)	53 (13.9)
	Acute lymphoblastic leukemia / acute undifferentiated leukemia)	24 (10.2)	3 (2.0)	27 (7.0)
	Hodgkin lymphoma	10 (4.2)	6 (4.1)	16 (4.2)
	Chronic myeloid leukemia	12 (5.1)	0 (0.0)	12 (3.1)
	Chronic lymphocytic leukemia	5 (2.1)	0 (0.0)	5 (1.3)
	Hemoglobinopathies	5 (2.1)	1 (0.7)	6 (1.6)
	Bone marrow failure	9 3.8)	0 (0.0)	9 (2.3)
	Inherited disorders	11 (4.7)	0 (0.0)	11 (2.9)
	Auto-immune diseases	1 (0.4)	1 (0.7)	2 (0.5)
	Other diseases	2 (0.8	3 (2.0)	5 (1.3)

Supplementary table 2: Symptoms at diagnosis

	Allogeneic N = 236 (%)	Autologous N = 146 (%)
Asymptomatic	20 (8.5)	14 (9.6)
Fever	154 (65.3)	103 (70.5)
Cough	129 (54.7)	80 (54.8)
Upper respiratory symptoms	58 (24.6)	48 (32.9)
Fatigue	99 (41.9)	63 (43.1)
Myalgia or arthralgia	33 (14.0)	25 (17.1)
Diarrhea	28 (11.9)	24 (16.4)
Vomiting	16 (6.8)	17 (11.6)
Oxygen requirement	76 (32.2)	56 (38.4)

Supplementary table 3: Interventions during management of COVID-19.

Given agent	n
Potential antivirals	
Remdesivir	14
Chloroquine/hydroxychloroquine	81
Lopinavir/ritonavir	44
Favipiravir	3
Antiinflammatory agents	
Corticosteroids	48
Tocilizumab	40
Anakinra	15
Ruxolitinib	4
Eculizumab	3
Siltuximab	2
Baricitinib	2
Mesenchymal stem cells	1
Other	
Convalescent plasma	5

Supplementary table 4: Univariate analyses

		overall survival		resolution		LRTD		ICU admission	
Variable		HR (95% C.I.)	p	HR (95% C.I.)	p	OR (95% C.I.)	p	OR (95% C.I.)	p
Age at covid	Continuous (10-yr effect)	1.34 (1.17-1.55)	<0.0001	0.94 (0.88-1.01)	0.09	1.36 (1.19-1.57)	<0.0001	1.09 (0.94-1.25)	0.2
Age at COVID (Allo only)	Continuous (10-yr effect)	1.30 (1.12-1.52)	0.0007	0.94 (0.87-1.02)	0.1	1.39 (1.17-1.64)	0.0002	1.14 (0.96-1.34)	0.1
Age at COVID (Auto only)	Continuous (10-yr effect)	1.56 (1.10-2.22)	0.01	0.95 (0.79-1.13)	0.6	1.57 (1.12-2.20)	0.009	0.95 (0.68-1.31)	0.7
Type of HSCT	Autologous	1.00		1.00		1.00		1.00	
	Allogeneic	0.95 (0.64-1.40)	0.8	0.89 (0.68-1.17)	0.4	0.95 (0.54-1.67)	0.9	1.00 (0.60-1.67)	1.0
Diagnosis	AML/ALL	1.00	0.4	1.00	0.02	1.00	0.6	1.00	0.6
	CML/MDS/MPN	1.45 (0.82-2.59)		0.78 (0.53-1.15)		1.37 (0.60-3.13)		1.40 (0.64-3.07)	
	NHL/Hodgkin/CLL	1.22 (0.71-2.12)		0.65 (0.45-0.94)		1.68 (0.76-3.72)		1.79 (0.72-4.43)	
	Other	0.50 (0.90-2.51)		1.10 (0.79-1.52)		1.31 (0.66-2.59)		1.16 (0.43-3.10)	
Time from most recent transplant to COVID	<1 year	1.00		1.00		1.00		1.00	
	≥1 year	0.67 (0.46-0.98)	0.04	1.30 (0.99-1.70)	0.06	1.07 (0.61-1.86)	0.8	0.56 (0.34-0.93)	0.02
Time from most recent transplant to COVID (Allo only)	< 1 year	1.00		1.00		1.00		1.00	
	>= 1 year	0.58 (0.35-0.94)	0.028	1.37 (0.98-1.91)	0.07	0.59 (0.29-1.23)	0.16	0.48 (0.25-0.92)	0.03
Time from most recent transplant to COVID (Auto only)	< 1 year					1.00		1.00	
	>= 1 year					2.77 (1.12-6.88)	0.03	0.71 (0.31-1.62)	0.4
Performance status (Karnofsky/Lansky)	10-point effect	0.77 (0.69-0.86)	<0.0001	1.14 (1.02-1.27)	0.02	0.84 (0.67-1.04)	0.1	0.86 (0.74-1.02)	0.08
Performance status (Karnofsky/Lansky) (Allo only)	10-point effect	0.75 (0.66-0.86)	<0.0001	1.24 (1.08-1.43)	0.003	0.85 (0.65-1.11)	0.23	0.81 (0.66-1.00)	0.04

Performance status (Karnofsky/Lansky) (Auto only)	10-point effect	0.81 (0.68-0.97)	0.02	0.98 (0.82-1.18)	0.9	0.80 (0.54-1.18)	0.2	0.97 (0.73-1.27)	0.8
ISI group	Low	1.00	0.0008	1.00	0.04	1.00		1.00	
	Intermediate-High	2.54 (1.47-4.40)		0.73 (0.54-0.99)		2.15 (1.16-4.01)	0.015	2.04 (1.10-3.80)	0.02
ISI group (Allo only)	Low	1.00		1.00		1.00		1.00	
	Intermediate-High	3.88 (1.82-8.29)	0.0005	0.58 (0.39-0.84)	0.004	2.22 (0.99-4.94)	0.052	2.17 (1.01-4.66)	0.046
Ongoing steroids (Allo only)	No	1.00		1.00		1.00		1.00	
	Yes	1.12 (0.65-1.95)	0.7	0.72 (0.51-1.01)	0.06	1.80 (0.84-3.86)	0.1	1.94 (1.01-3.71)	0.046
Ongoing IS (non-steroid) therapy (Allo only)	No	1.00		1.00		1.00			
	Yes	2.18 (1.03-4.63)	0.04	0.57 (0.39-0.83)	0.003	1.74 (0.78-3.90)	0.2	2.56 (1.01-6.47)	0.047
Acute or chronic GVHD (Allo only)	No	1.00		1.00		1.00		1.00	
	Yes	1.22 (0.73-2.05)	0.4	0.70 (0.50-0.99)	0.04	1.06 (0.51-2.19)	0.9	1.34 (0.70-2.57)	0.4
ICU	No	1.00		1.00					
	Yes	3.44 (2.30-5.16)	<0.0001	0.47 (0.32-0.70)	0.0002				
ICU (Allo only)	No	1.00		1.00					
	Yes	4.11 (2.43-6.94)	<0.0001	0.42 (0.25-0.70)	0.001				
ICU (Auto only)	No	1.00		1.00					
	Yes	2.72 (1.44-5.16)	0.002	0.56 (0.30-1.04)	0.07				
Preexisting lung pathology	No	1.00		1.00		1.00		1.00	
	Yes	1.33 (0.82-2.15)	0.25	0.70 (0.48-1.01)	0.05	1.40 (0.64-3.05)	0.4	1.44 (0.76-2.73)	0.3
Preexisting lung pathology (allo only)	No	1.00		1.00		1.0		1.00	

	Yes	1.15 (0.63-2.09)	0.7	0.86 (0.57-1.30)	0.5	1.35 (0.54-3.32)	0.5	1.33 (0.62-2.87)	0.5
Preexisting lung pathology (auto only	No	1.00		1.00		1.00		1.00	
	Yes	1.76 (0.78-3.96)	0.2	0.36 (0.15-0.84)	0.02	1.62 (0.34-7.87)	0.5	1.74 (0.54-5.66)	0.4
ANC	<500	1.00		1.00				1.00	
	≥500	0.49 (0.27-0.88)	0.018	1.53 (0.87-2.70)	0.1			0.99 (0.38-2.56)	1
ALC	<200	1.00		1.00		1.00		1.00	
	≥200	0.74 (0.41-1.33)	0.31	1.64 (1.02-2.66)	0.04	0.89 (0.33-2.42)	0.8	0.65 (0.30-1.40)	0.3
ALC (Allo only)	<200	1.00		1.00		1.00		1.00	
	≥200	0.71 (0.33-1.51)	0.4	2.03 (1.08-3.83)	0.03	1.06 (0.29-3.90)	0.9	0.82 (0.28-2.42)	0.7
Neutrophil/lymphocyte ratio	<median (3.25)	1.00		1.00		1.00		1.00	
	≥median (3.25)	0.74 (0.48-1.14)	0.17	0.99 (0.74-1.33)	1	1.74 (0.91-3.30)	0.09	2.08 (1.20-3.59)	0.009
Neutrophil/lymphocyte ratio (Allo only)	<median (3.25)	1.00		1.00		1.00		1.00	
	≥median (3.25)	0.59 (0.33-1.04)	0.07	0.86 (0.59-1.24)	0.4	1.49 (0.65-3.43)	0.3	1.75 (0.88-3.51)	0.1
Neutrophil/lymphocyte ratio (Auto only)								1.00	
								2.78 (1.12-6.95)	0.03
Lymphocyte/CRP ratio	<median (0.033)	1.00		1.00		1.00		1.00	
	≥median (0.033)	0.55 (0.35-0.86)	0.009	1.02 (0.76-1.37)	0.9	0.29 (0.14-0.61)	0.001	0.34 (0.19-0.61)	0.0003
Lymphocyte/CRP ratio (Allo only)	<median (0.033)	1.00		1.00		1.00		1.00	
	≥median (0.033)	0.89 (0.63-1.25)	0.5	1.09 (0.74-1.61)	0.6	0.25 (0.09-0.68)	0.007	0.49 (0.24-1.00)	0.05
Lymphocyte/CRP ratio (Auto only)	<median (0.033)	1.00		1.00		1.00		1.00	
	≥median (0.033)	0.88 (0.43-1.77)	0.7	0.86 (0.53-1.41)	0.6	0.35 (0.12-1.05)	0.06	0.13 (0.04-0.46)	0.002
Country	Spain							1.00	0.03
	Italy-UK							084 (0.41-1.69)	
	Other							1.80 (1.02-3.20)	

Supplementary figure 1. Number of patients with or without ongoing immunosuppression when diagnosed with COVID-19 split by time from HCT to COVID-19 diagnosis.

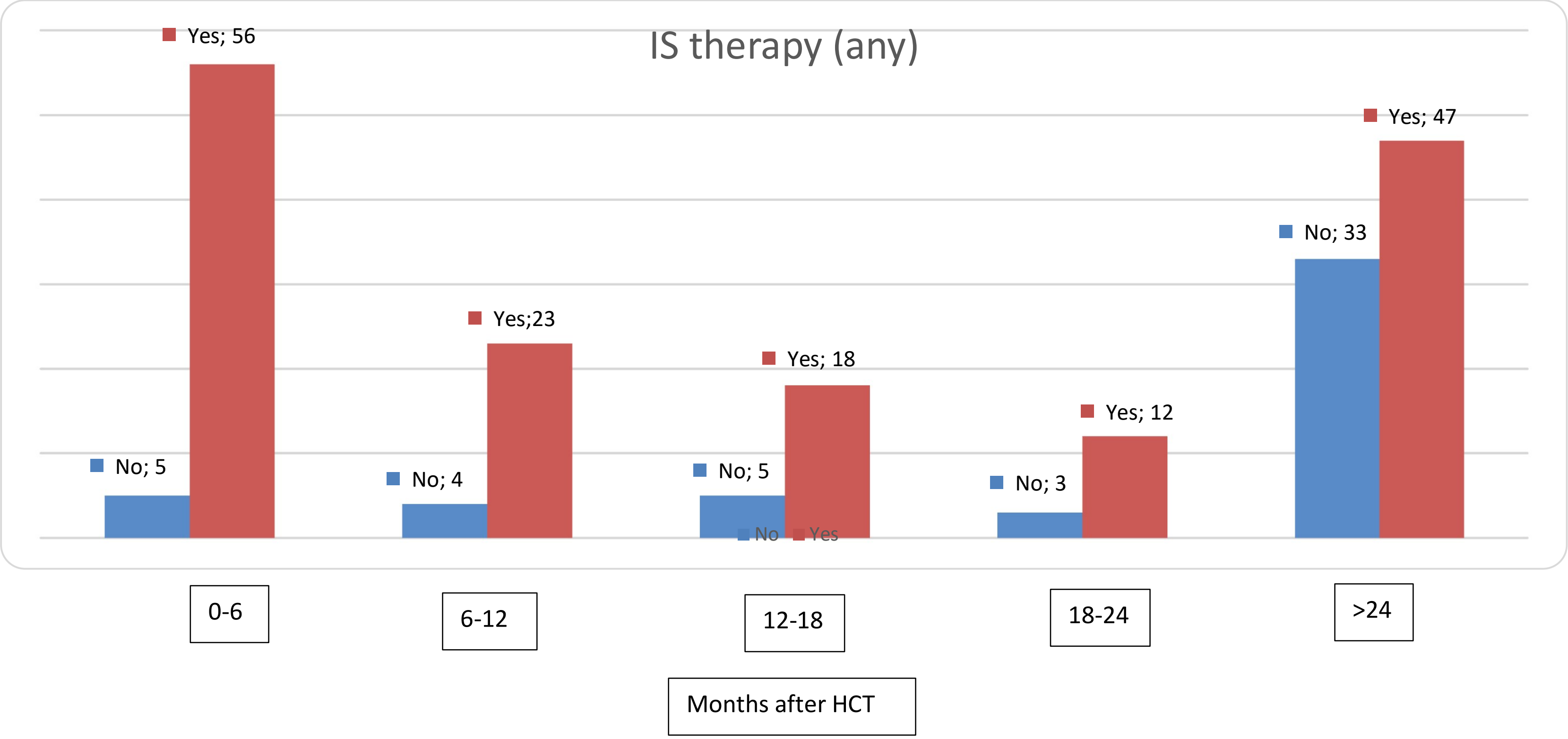

Supplement: Supplementary file 1 — Supplementary tables and figure [file 41375_2021_1302_MOESM1_ESM.pdf]
