# Supplementary material for: COVID-19 and stem cell transplantation; results from an EBMT and GETH multicenter prospective survey
Source: Leukemia. 2021 Jun 2;35(10):2885–94. doi: 10.1038/s41375-021-01302-5 (PMC8171362; doi:10.1038/s41375-021-01302-5)
Supplement: Supplementary file 2 — Contributors [file 41375_2021_1302_MOESM2_ESM.docx]

Contributing centers

Nicolaas Schaap, Nijmegen Medical Centre, Nijmegen, Netherlands;

Matthew Collin, Adult HSCT unit, Newcastle_Tyne, UK;

Antonio Campos, Inst. Português de Oncologia do Porto, Porto, Portugal;

Keith M. O. Wilson, Department of Haematology, Cardiff, UK;

Maria Jesús Pascual Cascon, Hospital Regional de Málaga, Malaga, Spain;

Rafael Duarte, Clinica Puerta de Hierro, Madrid, Spain;

Nabil Yafour, University Hospital of Oran, Oran, Algeria;

Zubeyde Nur Ozkurt, Gazi University Faculty of Medicine, Ankara, Turkey;

Joan Hendrik Veelken, Leiden University Hospital, Leiden, Netherlands;

Yves Beguin, University of Liege, Liege, Belgium;

Antonio Perez Martinez, Hospital Universitario La Paz, Madrid, Spain;

Victoria Potter, Kings College Hospital, London, UK;

Amir Ali Hamidieh, Children`s Medical Centre/Tehran University of Medical Sciences, Teheran, Iran;

Amal AlSeraihy, King Faisal Specialist Hospital and Research Centre, Riyadh, Saudi Arabia;

Wilfried Schroyens, Antwerp University Hospital (UZA), Antwerp_Edegem, Belgium;

Henrik Sengeloev, Bone Marrow Transplant Unit L 4043, Copenhagen, Denmark;

Emma Nicholson, Royal Marsden Hospital, London, UK;

Anjum Khan, Yorkshire Blood & Marrow Transplant Programme, Leeds, UK;

Yves Chalandon, Département d`Oncologie, Service d`Hématologie, Geneva, Switzerland;

Kristina Carlson, University Hospital, Uppsala, Sweden;

David Gallardo, Institut Català d`Oncologia, Girona, Spain;

Petr Sedlacek, University Hospital Motol, Prague, Czech Rep;

Matthias Klammer, St. George`s Hospital, London, UK;

Manuel Jurado Chacón, Hospital Univ. Virgen de las Nieves, Granada, Spain;

Eleni Tholouli, Manchester Royal Infirmary, Manchester, UK;

Javier López-Jiménez, Hospital Ramón y Cajal, Madrid, Spain;

Dries Deeren, AZ Delta, Roeselare, Belgium;

Bruno Lioure, Techniciens d`Etude Clinique suivi de patients greffes, Strasbourg, France;

Jörg Cammenga, University Hospital, Linkoeping, Sweden;

John Snowden, Sheffield Teaching Hospitals NHS Trust, Sheffield, UK;

Sonja Martin, Robert_Bosch_Krankenhaus, Stuttgart, Germany;

Johan Maertens, University Hospital Gasthuisberg, Leuven, Belgium;

Montserrat Rovira, Hospital Clinic, Barcelona, Spain;

Angus J M Broom, Western General Hospital, Edinburgh, UK;

Mercedes Colorado Araujo, Hospital U. Marqués de Valdecilla, Santander, Spain;

Francesco Onida, Fondazione IRCCS - Ca’ Granda, Milano, Italy;

Stig Lenhoff, Skanes University Hospital, Lund, Sweden;

Manos Nikolousis, Birmingham Heartlands Hospital, Birmingham, UK;

Francesco Lanza, Unità Operativa di Ematologia, Ravenna, Italy;

Rachel Protheroe, Bristol Royal Hospital for Children, Bristol, UK;

Juan Carlos Garcia-Ruiz, Hospital Universitario Cruces, Barakaldo, Spain;

Fergus Jack, Poole Hospital NHS Foundation Trust, Poole, UK;

Jose Luis Bello López, Hospital Clinico Universitario , S_de_Compostela, Spain;

Teresa Zudaire, Unidad de Ensayos Clínicos de Hematología Pabellón A, bajo., Pamplona, Spain;

Brenda E. Gibson, Royal Hospital for Children, Glasgow, UK;

Marta Gonzalez Vicent, Niño Jesus Children`s Hospital, Madrid, Spain;

Arnon Nagler, Chaim Sheba Medical Center, Tel_Hashomer, Israel;

Ipek Yonal-Hindilerden, Ýstanbul Tip Fakultesi, Istanbul, Turkey;

Josu de la Fuente, Division of Paediatrics, London, UK;

Domenico Russo, USD Trapianti di Midollo, Adulti, Brescia, Italy;

Mariagrazia Michieli, Centro di Riferimento Oncologico, Aviano, Italy;

Bruno Benedetto, S.S.C.V.D Trapianto di Cellule Staminali, Torino, Italy;

Xavier Poiré, Cliniques Universitaires St. Luc, Brussels, Belgium;

Prof. J.J. Cornelissen, Erasmus MC Cancer Institute, Rotterdam, Netherlands;

Sebastien Maury, Hôpital Henri Mondor, Creteil, France;

Paul Browne, Hope Directorate, Dublin, Ireland;

Claude Eric Bulabois, CHU Grenoble Alpes - Université Grenoble Alpes, Grenoble, France;

Ibrahim Yakoub-Agha, CHU de Lille, Lille, France;

Carlos Solano, Hospital Clínico de Valencia, Valencia, Spain;

Jan-Erik Johansson, Sahlgrenska University Hospital, Goeteborg, Sweden;

Manuel Abecasis, Inst. Portugues Oncologia, Lisboa, Portugal;

Lev Shvidel, Kaplan Medical Centre, Rehovot, Israel;

Tsila Zuckerman, Rambam Medical Center, Haifa, Israel;

Amjad Hayat, The Blood and Tissue Establishment, Galway, Ireland;

T. Netelenbos, Haga Hospital (Leyenburg), The_Hague, Netherlands;

Mareike Verbeek, Klinikum Rechts der Isar, Munich, Germany;

Achilles Anagnostopoulos, George Papanicolaou General Hospital, Thessaloniki, Greece;

Gwendolyn Van Gorkom, University Hospital Maastricht, Maastricht, Netherlands;

Paolo Corradini, University of Milano, Milano, Italy;

Fabio Benedetti, Policlinico G.B. Rossi, Verona, Italy;

Rik Schots, Universitair Ziekenhuis Brussel, Brussels, Belgium;

Jenny Byrne, Nottingham University, Nottingham, UK;

Aleksandr Kulagin, First State Pavlov Medical University of St. Petersburg, St_Petersburg, Russia;

Jose Rifón, Clínica Universitaria de Navarra, Pamplona, Spain;

Jose Antonio Pérez-Simón, Hospital Universitario Virgen del Rocío, Sevilla, Spain;

Adrian Bloor, Christie NHS Trust Hospital, Manchester, UK;

Sarah Lawson, Birmingham Children`s Hospital, Birmingham, UK;

Matthias Edinger, University Regensburg, Regensburg, Germany;

Luciano Wannesson, Ospedale Regionale Bellinzona e Valli, Bellinzona, Switzerland

Alicja Chybicka, Cape of Hope, Wroclaw, Poland;

Peter Albert Meyer, Stavanger University Hospital, Stavanger, Norway
